# Impaired activity-dependent neural circuit assembly and refinement in autism spectrum disorder genetic models

**DOI:** 10.3389/fncel.2014.00030

**Published:** 2014-02-07

**Authors:** Caleb A. Doll, Kendal Broadie

**Affiliations:** ^1^Department of Biological Sciences, Vanderbilt UniversityNashville, TN, USA; ^2^Kennedy Center for Research on Human Development, Vanderbilt UniversityNashville, TN, USA

**Keywords:** fragile X syndrome, synaptogenesis, synapse elimination, E/I ratio, optogenetics, *Drosophila*

## Abstract

Early-use activity during circuit-specific critical periods refines brain circuitry by the coupled processes of eliminating inappropriate synapses and strengthening maintained synapses. We theorize these activity-dependent (A-D) developmental processes are specifically impaired in autism spectrum disorders (ASDs). ASD genetic models in both mouse and *Drosophila* have pioneered our insights into normal A-D neural circuit assembly and consolidation, and how these developmental mechanisms go awry in specific genetic conditions. The monogenic fragile X syndrome (FXS), a common cause of heritable ASD and intellectual disability, has been particularly well linked to defects in A-D critical period processes. The fragile X mental retardation protein (FMRP) is positively activity-regulated in expression and function, in turn regulates excitability and activity in a negative feedback loop, and appears to be required for the A-D remodeling of synaptic connectivity during early-use critical periods. The *Drosophila* FXS model has been shown to functionally conserve the roles of human FMRP in synaptogenesis, and has been centrally important in generating our current mechanistic understanding of the FXS disease state. Recent advances in *Drosophila* optogenetics, transgenic calcium reporters, highly-targeted transgenic drivers for individually-identified neurons, and a vastly improved connectome of the brain are now being combined to provide unparalleled opportunities to both manipulate and monitor A-D processes during critical period brain development in defined neural circuits. The field is now poised to exploit this new *Drosophila* transgenic toolbox for the systematic dissection of A-D mechanisms in normal versus ASD brain development, particularly utilizing the well-established *Drosophila* FXS disease model.

## INTRODUCTION

The recent passing of David Hubel (September 22, 2013) occurs in the midst of a rich era of research into the activity-dependent (A-D) formation and refinement of neural circuitry during normal brain development and in neurodevelopmental disease states. Hubel and Wiesel’s pioneering studies on monocular deprivation and activity manipulations in the cat visual system (Hubel and Wiesel, 1959, 1962, 1970; Wiesel and Hubel, 1963) laid the foundation for our understanding of the A-D assembly and pruning of synaptic connections. All synapses formed through the reciprocal, highly orchestrated crosstalk between axons and dendrites face the bottleneck decision of elimination versus long-term maintenance and strengthening to form a stable partnership (dendrite stabilization review, Koleske, 2013; intrinsic dendrite development, Puram and Bonni, 2013). Although early synaptogenesis proceeds via largely activity-independent mechanisms, the refinement of synapses is a progressive, A-D process most active during the early-use critical periods of postnatal development, when synaptic arrays are most amenable to pruning and *de nova* additions (Hensch, 2004). Following this refinement period, A-D modulation is greatly reduced in the mature brain, except for maintenance of the synaptic plasticity underlying behavioral adaptation (Rice and Barone, 2000; rodent visual cortex, Nataraj and Turrigiano, 2011). Recent advances in biotechnology provide high-fidelity readouts of neural activity, as well as precise, non-invasive methods for the bidirectional manipulation of neural activity (Chen et al., 2013; Lin et al., 2013b), generating the means to study A-D developmental processes at a previously inconceivable level.

Autism spectrum disorders (ASDs) are defined by social interaction impairments (Abrahams and Geschwind, 2008), frequently accompanied by sensory hypersensitivity, cognitive deficits, and A-D seizures (Kim and Lord, 2012; Kim et al., 2013b). The improper development of neural circuitry likely lies at the heart of ASDs, particularly the A-D processes of solidifying appropriate synaptic connections and concomitantly pruning superfluous or incorrect connections (Zoghbi and Bear, 2012). The apparently diverse genetic bases of the wide spectrum of autism-related disorders makes genetic modeling a challenge (Sanders et al., 2012), but recent hypotheses suggest that the variety of genetic variants associated with ASDs may converge on a more manageable set of core molecular pathways (Murdoch and State, 2013). With this in mind, targeted mouse and *Drosophila* animal model systems harboring deficiencies in ASD-linked human genes often show comparable phenotypic and behavioral defects to human patients (Hagerman et al., 2009; van Alphen et al., 2013). Among the strongest primary research contributions have come from models of fragile X syndrome (FXS), a monogenic disorder that is the leading heritable contributor to the autism spectrum (Harris et al., 2008; McBride et al., 2012). In both mouse and *Drosophila* FXS models, there is clear and consistent evidence that the causal fragile X mental retardation protein (FMRP) is directly activity-regulated and in turn regulates A-D processes of neural circuit assembly and refinement (Wang et al., 2010a,b; Wondolowski and Dickman, 2013). Preclinical studies with these animal models have already advanced to a number of human clinical trials [e.g., metabotropic glutamate receptor (mGluR) therapeutics], and groundbreaking tools to assess and manipulate A-D synapse and circuit development show great promise toward major breakthroughs in ASD therapeutic intervention strategies (Akerboom et al., 2013; Paz et al., 2013; Sukhotinsky et al., 2013).

In this review article, we seek to highlight recent advances in our understanding of A-D synaptic development in the normal and ASD brain, particularly focused on recent work from mouse and *Drosophila* genetic models. We will only mention is passing electrophysiological investigations of synaptic plasticity at maturity, which is the focus of many excellent reviews (Malenka and Bear, 2004; Nelson and Turrigiano, 2008; Castillo et al., 2011). Likewise, the broad genetic and molecular details of A-D neural circuit assembly have recently been presented elsewhere (Flavell and Greenberg, 2008; West and Greenberg, 2011; Ebert and Greenberg, 2013). Our main focus will be on the A-D basis of ASDs, and particularly on FXS, as the leading heritable contributor to this neurodevelopmental disease condition (Hagerman and Hagerman, 2002). Starting with a brief review of normal experience-dependent synaptic changes (Part 1), we will then focus on correlates between the ASD disease state and A-D circuit formation (Part 2), and finally finish with a detailed review of the recent technological advances for the manipulation and monitoring of A-D processes (Part 3) during neural circuit development.

## PART 1: NORMAL ACTIVITY-DEPENDENT NEURODEVELOPMENT

**FIGURE 1:**
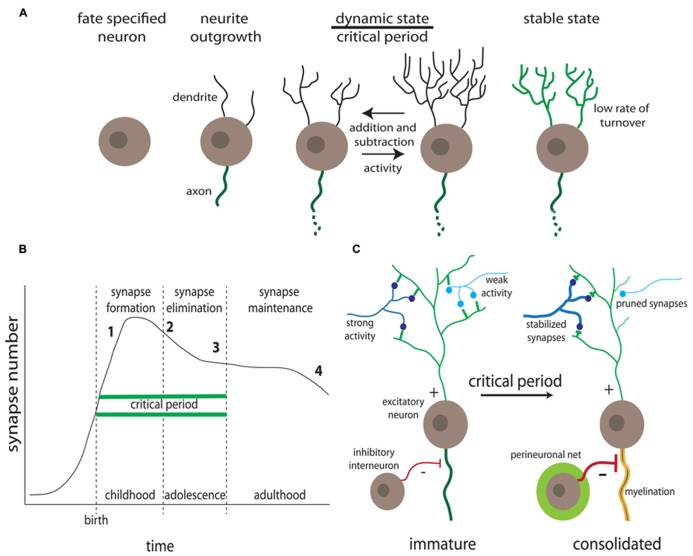
**Activity-dependent synaptic remodeling during critical periods.** **(A)** Progression of dendrite development. Highly dynamic dendrites occur in the A-D critical period prior to consolidation of relatively stable mature connections (Kreutz and Sala, 2012). **(B)** Synapse number changes as a function of age. Defects in synaptogenesis (formation, pruning, and stabilization) correlate with onset of specific neurological disorder classes, including intellectual disability (1), ASDs (2), schizophrenia (3), and Alzheimer’s disease (4) (for example, Penzes et al., 2011). **(C)** Activity-dependent synapse changes occur during the critical period, in which synaptic partnerships are solidified or dissolved prior to consolidation, coinciding with inhibitory interneuron innervation, progressive myelination and the formation of extracellular perineuronal nets (Hensch, 2003).

Hebb (1949) theorized that neural activity would code neural circuit connectivity through a mechanism of coincident synapse elimination and consolidation. This theory was first tested in the cat visual cortex, with the first visual response recordings made in the 1950s (Bishop and Clare, 1951, 1952, 1955; Clare and Bishop, 1954; Jung, 1958), coincident with the pioneering work of Kuffler (1953) defining ganglion cell specificity/organization and producing some of the first primary evidence of higher order processing. Kuffler’s students went on to establish the principles of A-D mechanisms (**Figure 1A**), including Horace Barlow’s characterization of selectivity and lateral inhibition in the frog retina (Barlow, 1953a,b), and David Hubel and Torsten Wiesel’s work on the basis of A-D (and later experience-dependent) synaptic development in the cat retinal system. Hubel and Wiesel first demonstrated that individual striatal cortical neurons (primary visual cortex) respond preferentially to slits of light (Hubel and Wiesel, 1959), providing a mechanism by which cortex organization enables higher order perception (Hubel and Wiesel, 1962). Their subsequent studies using monocular deprivation revealed profound changes in cortex development, with active-pathway axons from the lateral geniculate nucleus (LGN) dramatically out-competing inactive axons for cortex innervation of striatal cortical neurons (Wiesel and Hubel, 1963). The LGN innervated by the monocularly deprived retinal axons was also thinner, demonstrating a sensory experience-dependent restructuring of the developing neural circuit (Wiesel and Hubel, 1963).

Hubel and Wiesel went on to perform an extended series of A-D developmental studies, establishing a critical period for visual cortical development in kittens, and demonstrating that adult cats show no comparable experience-dependent morphological or electrophysiological changes (Wiesel and Hubel, 1963, 1965a,b; Hubel and Wiesel, 1970; Wiesel, 1982; Cohen and Greenberg, 2008). Following these pioneering studies, A-D morphological changes were similarly revealed in other areas of the sensory cortex. As one example, upon trimming whiskers in specific rows, axonal projections in the rat somatosensory cortex were reduced from non-deprived columns into deprived columns (axons from column A generally innervate column B; if the B column is deprived of input, the A axon receives no postsynaptic response and collapses), and increased horizontal axonal projections between non-deprived columns (Broser et al., 2008). Whisker trimming on the rat’s snout from birth leads to a smaller contralateral motor area that evokes abnormal motor activity, a phenomenon not seen in adult rats (Huntley, 1997), again indicating a transient developmental window. Neuromuscular junction (NMJ) innervation is another classic system for studying A-D remodeling (Schuster, 2006). Motor axons compete to target individual muscle fibers during the early-use neonatal period (Sanes and Lichtman, 1999), and NMJ development in the first couple of neonatal weeks displays a progression of A-D synapse elimination, functional reinforcement, and eventual structural consolidation (Lichtman and Colman, 2000; Walsh and Lichtman, 2003). Consistently, impeding neural activity results in slowed synaptic refinement in the mouse neuromusculature, and enhancing activity increases the rate of development (Thompson, 1985).

Humans show similar mechanisms of A-D neural circuit development (**Figure 1B**). For example, the auditory cortex displays an early age critical window of experience-dependent maturation, with professional musicians developing asymmetric brain features when exposed to music before the age of 7. Specifically, development of absolute pitch correlates with a larger left planum temporale (Schlaug et al., 1995), and enlarged cortical representation of the left hand in dexterous string players (Elbert et al., 1995; Schlaug et al., 1995). Auditory cortical development may actually represent a more extensive (or indefinite) critical period, as compared to other sensory modalities (Kilgard and Merzenich, 1998; Chang and Merzenich, 2003). This relative extension may result from a late peak of parvalbumin-expressing (PV+) interneurons, as described in ferret brain development (Gao et al., 2000). The emergence of these inhibitory interneurons is progressive (Honig et al., 1996) and vital for the proper formation of cortical circuits (**Figure 1C**; Powell et al., 2012). The auditory critical period is not open ended, however, as childhood ear infections leading to long-term deficits in auditory perceptual acuity can occur if not treated before the age of 7 (Popescu and Polley, 2010). These select examples illustrate normal A-D development within single sensory modalities. ASD symptoms may manifest through faulty A-D development in a number of sensory systems, with impairments of higher-order cognition circuitry developing after formation of primary sensory circuitry (Belmonte et al., 2004; Geschwind and Levitt, 2007). Although ASD diagnoses focuses on higher order cognitive tasks such as social communication, language and cognitive development, and repetitive behaviors (Zwaigenbaum et al., 2013), precursor deficits in primary sensory processing are characteristic (Marco et al., 2011). A more detailed discussion of ASD phenotypes is presented in Part 2.

### CRITICAL PERIODS OF NEURAL CIRCUIT DEVELOPMENT

The highly dynamic nature of synaptic connectivity is largely a transient feature of neurodevelopment: long-term imaging of dendritic spines in adult mice reveals that most mature synapses are relatively stable (Grutzendler et al., 2002). The critical window (or critical period) theory has emerged to explain the decline in synaptic dynamics as the brain develops, and as a mechanistic foundation toward understanding ASD disease states (**Figure 1**; Hensch, 2004). A critical period defines a temporary developmental window of heightened sensitivity to sensory stimuli, which drive connectivity changes (Holtmaat and Svoboda, 2009), with A-D modulation reduced after the window passes, as reflected by the decrease in spine turnover as the brain matures (Trachtenberg et al., 2002; Holtmaat et al., 2005; Zuo et al., 2005). The key critical period hallmarks include (1) competition between circuit elements, (2) neural activity regulation, (3) structural solidification of maintained connections, (4) sharply-defined experience-dependent window, (5) variable/hierarchical timing and duration of windows across systems, (6) a diversity of molecular mechanisms underlying A-D modulation, (7) emergence and connectivity of inhibitory neurons, (8) attention/motivation by aminergic and cholinergic modulation, and (9) the potential for reactivation in adulthood (**Figure 1**; Hensch, 2004). Synaptogenesis underlies these hallmarks in critical periods, and takes place sequentially through initial axonal/dendritic outgrowth, excess formation of synapses, and subsequent pruning through A-D maturation (Katz and Shatz, 1996; West and Greenberg, 2011). Although synapse regulation continues throughout life (Holtmaat et al., 2005; Grillo et al., 2013), peak synaptogenesis occurs during early postnatal life (Pan and Gan, 2008).

The terminal periods of critical windows coincide with other hallmarks of neurodevelopment (**Figure 1C**). These include the progressive myelination of nerve fibers, which is a process essential for cortical function; mice caged in isolation 2 weeks after weaning show reduced myelin and diminished cortical function (Makinodan et al., 2012). Importantly, myelination is also delayed in FXS (Pacey et al., 2013). Also relevant to ASD disease states is the emergence and maturation of local inhibitory (I) interneurons coincident with the end of critical periods, to provide balance to young excitatory (E) circuits (**Figure 1C**; Hensch, 2004, 2005). The correct development of E/I ratio is critical to neural circuit output, and defects in the E/I ratio balance is a leading candidate mechanism for explaining the emergence of ASD disease states (Gatto and Broadie, 2010). Both hyperexcitation and hypoinhibition are recurring themes in numerous ASD models (Casanova, 2006). More generally, genetic disruption of cortical interneuron development causes regional GABAergic deficits, epilepsy and ASD-like behavioral changes in mice (Powell et al., 2003). As one example, mice deficient for the axon guidance receptor *neuropilin 2* display reduced cortical interneuron numbers and are more prone to seizure following neuronal excitation (Gant et al., 2009). Thus, the critical period theory must include the temporally phased regulation of first excitatory and then inhibitory synapses, such that A-D synapse selection generates the appropriate E/I synapse ratio balance. There are other hallmarks of critical period cessation, including the A-D establishment of the perineuronal network, a matrix of chondroitin sulfate proteoglycans (**Figure 1C**; Ye and Miao, 2013). Roles of glycan modifications in ASD models will be further discussed below in Part 2.

### ACTIVITY-DEPENDENT SYNAPSE MECHANISMS IN *Drosophila*

Synaptic ultrastructure and function is remarkably conserved across species, for example, comparing mammalian brain glutamatergic synapses to *Drosophila* NMJ glutamatergic synapses (Schuster, 2006). Moreover, the underlying molecular elements of synapses are similarly extremely well conserved, allowing mutually complementary studies in animals ranging from rodents to flies (Featherstone and Broadie, 2000; Koles and Budnik, 2012). Most A-D work in *Drosophila* has focused on axonal (presynaptic) development (Rohrbough et al., 2003), whereas most comparable work in mouse has focused on dendritic (postsynaptic) spines. However, the *Drosophila* NMJ postsynaptic domain is well described and clearly subject to extensive A-D remodeling. It contains two functional classes of ionotropic glutamate receptors (iGluRs; Marrus et al., 2004) and a single metabotropic glutamate receptor (DmGluRA; Bogdanik et al., 2004). Postsynaptically, iGluRs are trafficked and stabilized downstream of A-D mechanisms (Thomas and Sigrist, 2012), and *in vivo* imaging has shown that presynaptic release of dense core vesicles is A-D, with potentiation of release dependent on Ca^2^^+^ influx and CaMKII (Ca^2^^+^/calmodulin-dependent protein kinase II) activation (Shakiryanova et al., 2007). DmGluRA nulls show increased A-D facilitation and decreased synaptic boutons of increased size, suggesting the receptor acts as an activity monitor controlling both synapse function and structure (Bogdanik et al., 2004). DmGluRA loss leads to increased expression of iGluRs, and DmGluRA over-expression leads to decreased iGluRs (Pan and Broadie, 2007), demonstrating a tight regulation of postsynaptic receptor composition as an activity response mechanism. These A-D processes in the postsynaptic domain are directly impacted in the *Drosophila* FXS disease model as discussed below in Part 2.

The *Drosophila *NMJ is a particularly dynamic synaptic structure during early development, with A-D growth modulated during larval crawling behavior and mediated via glutamatergic neurotransmission (Schuster, 2006). Live imaging shows NMJ growth proceeds by a variety of mechanisms: stretching of existing boutons and insertion of new boutons in between, adding new boutons to the end of an existing strand, *de novo* addition and branch formation from existing boutons (Zito et al., 1999). A crude method to increase NMJ transmission is through chronic rearing at 29–30°C, which results in accelerated synapse growth (Sigrist et al., 2003; Zhong and Wu, 2004). Spaced depolarization via high K^+^ saline application leads to the rapid extension and retraction of short filopodia, and the formation of synaptic boutons (Ataman et al., 2006). Reduced membrane excitability via inward current mutants (*paralytic* Na^+^ channel) or other Na^+^ channel loss-of-function (*tipE* or *mle*^nap-ts1^) leads to improper synaptic refinement (Jarecki and Keshishian, 1995; White et al., 2001), with significantly smaller synaptic boutons (Lnenicka et al., 2003). Acute depolarization of the NMJ leads to the formation of “ghost boutons,” that initially lack presynaptic active zones and postsynaptic iGluRs, which appear on a timescale of hours (Ataman et al., 2006, 2008). Generation of genetically targetable channels, such as the voltage-dependent UAS-EKO and UAS-Kir2.1 (Baines et al., 2001; Paradis et al., 2001), and the constitutively open UAS-dORK potassium channel shunt (White et al., 2001) allow a more precise dissection of the effects of activity regulating NMJ synaptic morphology. Recent advances in bioengineering have taken advantage of channel variants for genetically targeted hyperexcitation in *Drosophila*, including the *transient receptor potential* (TrpA1; Hamada et al., 2008) and TrpM8 thermogenic channels (Peabody et al., 2009), the constitutively active NaChBac channel (Nitabach et al., 2006), the expanding family of channelrhodopsin (ChR2) variants (Schroll et al., 2006; Ataman et al., 2008), and the hyperpolarizing eNpHR3.0 channel (Inada et al., 2011). These new methods will be more fully described below in Part 3.

Beyond neurotransmission *per se*, proper formation of the *Drosophila* NMJ entails other A-D *trans*-synaptic signaling mechanisms. Wnt signaling via wingless (Wg; Packard et al., 2002; Korkut and Budnik, 2009) functions downstream of activity (high K^+^ depolarization, ChR stimulation) to regulate both structural and functional development (Ataman et al., 2008; Korkut et al., 2009). Similarly in mammals, activity regulates *Wnt2* transcription, which stimulates dendritic arborization in hippocampal cultures (Wayman et al., 2006). Bone morphogenetic protein (BMP) signaling via glass bottom boat (Gbb; McCabe et al., 2003; Keshishian and Kim, 2004) leads to NMJ stabilization through LIM kinase 1 activity, preventing retraction and synapse loss (Eaton and Davis, 2005). Recent use of *Shaker* K^+^ channel mutants (or raising temperature to 30°C) to increase excitability, shows that retrograde BMP signaling is required for A-D NMJ growth and maturation (Berke et al., 2013). Heparan sulfate proteoglycan (HSPG) co-receptors of such signaling ligands (Ren et al., 2009), including Dally-like protein (Dlp) and Syndecan (Sdc), play important roles in NMJ synaptogenesis (Johnson et al., 2006; Dani et al., 2012). Importantly, HSPGs interact closely with FMRP to modulate *trans*-synaptic signaling in the *Drosophila* FXS disease model (Friedman et al., 2013), suggesting a link to A-D processes. Laminin A (LanA) is another extracellular synaptic protein of interest that is downregulated in response to activity to regulate synaptic architecture: LanA expression is inversely correlated with NMJ size, and is regulated by larval crawling activity, synapse excitation, postsynaptic response, and Wnt signaling (Tsai et al., 2012). These A-D processes in the presynaptic domain are directly impacted in the *Drosophila* FXS disease model as discussed below in Part 2.

Although A-D development has been explored at length at the *Drosophila* NMJ, more limited studies have examined A-D mechanisms in the brain, mainly focusing on the mushroom body (MB) learning and memory center (Zars et al., 2000; Margulies et al., 2005). These studies have been enhanced by recent generation of more targeted Gal4 drivers and new optogenetic tools allowing cell-autonomous, single-cell resolution dissection of A-D mechanisms in normal and disease states (Chiang et al., 2011). Using the mosaic analysis with a repressible cell marker (MARCM) clonal technique (Lee and Luo, 2001), characterization of MB axons shows critical period development at the level of individual cells: synaptic branches display significant A-D pruning during the early-use period following eclosion, but become relatively static at maturity (Tessier and Broadie, 2008). Importantly, sensory deprivation (SD) elevated synaptic branch number during this critical period, whereas activity depolarization of single-cell MARCM clones by ChR2 optical stimulation significant decreased synaptic branching during this same critical period (Tessier and Broadie, 2008). Similarly, recent work silencing olfactory sensory neurons (via UAS-Kir2.1; Limb3b-Gal4 or UAS-DorK; Limb3b) lead to immature axonal morphology, including broad axon terminals and multiple filopodia (Prieto-Godino et al., 2012). Silencing of a limited subset of projection neurons innervating the MB (UAS-dORK1.deltaC; Mz19-Gal4) leads to increased size, number, and active zone density of axon terminals within the microglumeruli of the MB calyx (Kremer et al., 2010). These A-D processes in the brain MB learning/memory center are directly impacted in the *Drosophila* FXS disease model, as discussed below in Part 2.

Information on A-D mechanisms regulating dendrites in the *Drosophila* genetic model is more limited, but this research focus is rapidly expanding. For example, motor neuron dendrite structural development has been shown to be regulated downstream of high K^+^ depolarization (Hartwig et al., 2008; Zwart et al., 2013). Moreover, a role for synaptic activity in dendritic remodeling has been shown via targeted transgenic tetanus toxin expression (UAS-TNT; Sweeney et al., 1995) blocking neurotransmitter release from cholinergic interneurons (Cha-Gal4; UAS-TNT) leads to increased dendritic structural complexity (Tripodi et al., 2008). Dendritic refinement in serotonergic neuron pupal development is also modulated by activity: hyperpolarization via UAS-Kir2.1 caused increased dendritic length, which was proposed to be due to A-D Wnt/Wg signaling with a pro-retraction role in sensory-input dendritic refinement (Singh et al., 2010). Similarly, the silencing of olfactory sensory neurons (via UAS-Kir2.1; Orco-Gal4) led to enhanced dendritic occupancy of the antennal lobe by projection neurons (Prieto-Godino et al., 2012). However, it is also clear that A-D differences may be found across different neuronal types or developmental stages. For example, increased firing of RP2 motor neurons caused by dominant negative *Shaker *and *eag* K^+^ channel mutations resulted in increased dendritic complexity, whereas Kir2.1 silencing resulted in decreased dendritic structure (Timmermans et al., 2013). Moreover, constitutively active CaMKII also led to increased dendrite length and branching (Timmermans et al., 2013). Use of the temperature-gated TrpA1 channel to activate neuron firing demonstrated that MN5 flight motor neuron dendrites respond to activity differently over time: increased activity before pupal day 6 caused decreased dendritic branching (Vonhoff et al., 2013), whereas increased activity later in development caused increased branching (Duch et al., 2008), again suggesting differential A-D critical periods.

Mammalian models of dendritogenesis display similar A-D mechanisms to those characterized above in *Drosophila*. For example, increased neural activity and glutamatergic signaling led to dendritic spine outgrowth (Jontes and Smith, 2000; Antar et al., 2006), and spine turnover rates in young mice were shown to be sensory experience-dependent (Trachtenberg et al., 2002). Long-term SD through whisker trimming led to dendritic spine pruning that was more prominent in young mice (Zuo et al., 2005), and spine synapse densities changed upon rearing or training in enriched environments (Greenough et al., 1985; Beaulieu and Colonnier, 1987; Moser et al., 1995). Spaced depolarization of hippocampal neurons in culture led to extension of new spines, a process correlated with A-D MAPK activation (Wu et al., 2001). Recent advances in live imaging have elegantly provided *in vivo* evidence of A-D dendritic spine dynamics (Alvarez and Sabatini, 2007; Holtmaat and Svoboda, 2009). As one example, the immediate early gene Arc/Arg3.1 functions to eliminate surplus climbing fibers (CF) onto Purkinje cell synapses in the cerebellum, a process that is accelerated with ChR2 depolarizing stimulation for 2 days and suppressed by targeted CF knockdown of voltage-gated Ca^2^^+^ channels (Mikuni et al., 2013). Limited regions of the adult brain remain amenable to similar changes, for example, hippocampal spine density increased in adult rats following spatial learning (Moser et al., 1995), and multiphoton imaging of dendritic spines during mGluR-induced long-term depression (LTD) showed spine shrinkage and spine elimination that persisted for up to 24 h (Ramiro-Cortes and Israely, 2013), but in general these synaptic dynamics are confined to critical periods of synaptogenesis during defined developmental windows.

### ACTIVITY-DEPENDENT TRANSCRIPTIONAL AND TRANSLATIONAL REGULATION OF SYNAPTOGENESIS

Activity-dependent gene transcription clearly leads to developmental changes in synaptic connectivity. Mouse studies of the transcriptional regulator methyl CpG binding protein 2 (MeCP2) are one elegant example, with knock-in mouse lacking the *neuronal activity-induced phosphorylation* (NAIP) sequence showing increased excitatory synaptogenesis (Li et al., 2011). MeCP2 is phosphorylated in response to activity and subsequent Ca^2^^+^ influx (CaMKII-dependent), leading to regulation of dendritic branching, spine morphogenesis, and A-D induction of brain-derived neurotrophic factor (BDNF) transcription (Zhou et al., 2006). Importantly, MeCP2-deficient mice exhibit delayed maturation of cortical synaptogenesis and neuronal architecture defects (Fukuda et al., 2005), and human MeCP2 mutations are causally associated with the ASD Rett syndrome (Amir et al., 1999). Indeed, many ASD candidate genes are expressed synaptically to modulate synapse function/morphology, and are directly regulated by synaptic activity (Zoghbi and Bear, 2012). Calcium influx has a profound impact on gene transcription (Greer and Greenberg, 2008). As one example, A-D Ca^2^^+^ influx leads to dephosphorylation of myocyte enhancing factor 2 (MEF2) by calcineurin, causing dissociation with histone deacetylases, CBP recruitment and ultimately, transcription-dependent synapse elimination (Flavell et al., 2006; Barbosa et al., 2008; Pulipparacharuvil et al., 2008). MEF2 activation also leads to suppression of excitatory synapse number via Arc (Flavell et al., 2006; Flavell and Greenberg, 2008), perhaps through Arc-mediated AMPA receptor internalization (Niere et al., 2012). This MEF2-regulated synapse elimination has been correlated with the acquisition of learning and memory abilities (Barbosa et al., 2008), such as those impacted in ASD disease states. Activity similarly regulates cAMP response element-binding protein (CREB), serum response factor (SRF), FBJ murine osteosarcoma viral oncogene (Fos; Greenberg et al., 1986), and neuronal PAS domain protein 4 (NPAS4; Lin et al., 2008), leading to the A-D transcriptional regulation of synaptic proteins, including ASD-associated BDNF, Arc, and ubiquitin-protein ligase E3A (Ube3A; Cohen and Greenberg, 2008; Greer and Greenberg, 2008). CREB and NPAS4 transcriptional activity, via BDNF A-D activation, also leads to a reduced number of inhibitory synapses on excitatory neurons (Hong et al., 2008; Lin et al., 2008), suggesting roles in the developmental regulation of E/I ratio.

Activity-dependent localized synaptic translation permits a rapid and synapse-specific response, which is particularly important in governing the multitude of differentially active synapses occurring at a distance from the cell body. RNA-binding proteins and translational regulation have been demonstrated in both axonal growth cones and mature axons (Hornberg and Holt, 2013), ostensibly permitting local protein production in presynaptic boutons. Highly motile RNA granules containing inactive ribosomes (Krichevsky and Kosik, 2001; Elvira et al., 2006), suggest neurons have evolved mechanisms to bypass translation initiation locally at the synapse (Costa-Mattioli et al., 2009; Batish et al., 2012). Assays of local translation using ribopuromycylation to visualize ribosomes associated with nascent peptide chains (David et al., 2012) demonstrate that mRNAs are transported alongside paused polyribosomes at hippocampal synapse, thereby bypassing the rate-limiting step of translation initiation (Graber et al., 2013). Importantly, these polyribosomes co-localize with RNA-binding FMRP and Staufen 2 (Antar et al., 2005; Elvira et al., 2006; Napoli et al., 2008; Darnell et al., 2011; Lebeau et al., 2011), and defects in A-D translational control can lead to ASD states, with unregulated translation causing synaptic impairment driving behavioral dysfunction (Santini et al., 2013). This topic will be explored at length in Part 2.

The strongest link between translation control and A-D synaptogenesis is the RNA-binding FMRP, which regulates translational initiation (Napoli et al., 2008), mRNA transport (Bassell and Warren, 2008), and translational elongation of mRNAs encoding synaptic proteins (Darnell et al., 2011). FMRP is strongly upregulated during critical periods of neural circuit refinement, where it associates with mobile RNA granules in dendrites, spines, filopodia, and growth cones that translocate in response to the level of neuronal activity (Antar et al., 2005; Cook et al., 2011). Importantly, FMRP is positivity upregulated by neuronal activity (Antar et al., 2004; Gabel et al., 2004; Tessier and Broadie, 2008; Wang et al., 2008b), and regulates multiple A-D processes including synapse elimination (Pfeiffer et al., 2010). Studies at the *Drosophila* NMJ first demonstrated that FMRP negatively regulates cytoskeletal targets, including the MAP1B/Futsch (Zhang et al., 2001) mediator of microtubule-associated synaptic growth (Roos et al., 2000). Interestingly, A-D transcriptional and translational control are linked through FMRP, as the activity of MEF2 in synapse elimination is wholly dependent on FMRP function, and occurs cell autonomously in the postsynaptic neuron (Pfeiffer et al., 2010). In *Fmr1* knockout (KO) hippocampal culture, acute expression of FMRP (via timed transfection) at an early postnatal period leads to synapse growth, but FMRP during the second postnatal week led to suppression of synapse formation (Zang et al., 2013). Interestingly, MEF2 activity is progressively increased upon depolarization (via high K^+^ treatment) over the same developmental period (Zang et al., 2013). These recent studies highlight both the impact of FMRP on synaptic growth and the importance of developmental timing within critical periods of development. In the following section (Part 2) we will elaborate the molecular details of FXS and other ASDs, highlighting A-D changes in the development of synaptic connectivity.

## PART 2: ACTIVITY-DEPENDENT MECHANISMS IN ASD DISEASE STATES

For ASD diagnosis, children must display three core symptoms before 3 years of age: (1) atypical social behavior, (2) disrupted verbal/non-verbal communication, and (3) unusual patterns of restricted interests or repetitive behaviors (Geschwind and Levitt, 2007). It has been proposed that a “disconnect” between brain regions involved in higher-order associations lies at the root of these ASD behaviors (Frith, 2004; Courchesne and Pierce, 2005; for historical context, Geschwind, 1965a,b). For example, this disconnect may occur between a pair of higher-order association cortices (or several such centers), which represent input from multiple sensory modalities in cortical space (Geschwind and Levitt, 2007). More evidence for this disconnect theory comes from prefrontal cortex and anterior cingulate disconnection for joint attention (foundation of language and social behavior; Mundy, 2003), and demonstrated disconnect in functional magnetic resources imaging (fMRI) studies (Koshino et al., 2005). The root of this hypothesis is based on the hierarchical development of cortical circuitry (LeBlanc and Fagiolini, 2011; i.e., disrupted development of the initial architecture, e.g., via shifts in critical periods) results in faulty substrates for subsequent A-D mechanisms that are crucial for reorganization, pruning, and solidification of synapses within circuits. Primary sensory cortices develop progressively, with critical periods that are variable in time and often non-overlapping (Kroon et al., 2013). Furthermore, individual modalities develop progressively, as the critical periods of rodent somatosensory cortex begin at the subcortical level and then progress to cortical levels (Feldmeyer et al., 2013). The “missed window” theory of ASDs may help explain the root of auditory, visual, and somatosensory dysfunction in information processing, which drives the socialization and communication deficits defining ASDs (LeBlanc and Fagiolini, 2011). In terms of basic brain architecture, ASDs may emerge through faulty subcortical development, which precedes thalamic as well as subsequent cortical development (Kroon et al., 2013). Evidence for this theory includes early incidence of motor developmental delay, social impairment, and epileptic seizures (Zoghbi and Bear, 2012).

The hierarchical model of ASD brain development is built upon evidence that alterations in primary sensory modalities underlie higher order cognition defects. Broadly speaking, these primary sensory alterations appear to lessen in severity with age, although the severity of primary sensory impairments clearly correlates with the degree of social interaction impairment (Ben-Sasson et al., 2009; Simmons et al., 2009). Neuronal activity is essential for circuit development (Lendvai et al., 2000; Spitzer, 2006), and this activity is both intrinsically generated (Golshani et al., 2009; Rochefort et al., 2009) and sensory derived, as shown in primary visual cortex (Siegel et al., 2012). However, this A-D component of circuit development is obviously built upon genetic foundations, and several hundred genes are associated with ASDs (State and Sestan, 2012). Clinical characterizations of ASDs are highly suggestive of A-D synaptic defects (Toro et al., 2010; Ebert and Greenberg, 2013). For example, FXS patients display hypersensitivity to numerous sensory stimuli (Miller et al., 1999), as well as abnormal sensory gating in prepulse inhibition trials (Hessl et al., 2009). In addition, attention deficit/hyperactivity disorder (Murray, 2010) and developmentally transient epilepsy are also associated with FXS (Musumeci et al., 1999; Berry-Kravis, 2002) strongly indicating a core A-D impairment.

Although it is understandably difficult to model ASD behaviors in animals, several recent studies demonstrate inventive ways to address this essential issue. For example, *Fmr1* KO mice display many behavioral disruptions similar to human FXS patients, including susceptibility to audiogenic seizure, hyperactivity, learning and memory deficits, and social interaction abnormalities (Kooy, 2003; Bear et al., 2004; Hagerman et al., 2009). Some studies report that *Fmr1* KO mice show increased anxiety-related activity during social interaction (Mines et al., 2010), whereas other studies show *decreased* anxiety in open field studies (Michalon et al., 2014). It is quite clear that behavioral phenotypic effects are heavily dependent on genetic background (Spencer et al., 2011). However, *Fmr1* KO mice in the C57 background show consistent impairments in social interaction behaviors (Baker et al., 2010). Research in mice has also focused on single sensory modalities, showing that *Fmr1* KO mice display altered auditory processing (Rotschafer and Razak, 2013). Although higher cognition is difficult to test in mice, recent research using a touch-screen operant conditioning paradigm (which facilitates a conflict between sensory-driven and task-dependent signals, thereby increasing cognitive load) demonstrates that *Fmr1* KO mice display defects in learning under heavy cognitive demand (Dickson et al., 2013). The *Drosophila* FXS model similarly shows disruptions closely resembling human FXS patients, including hyperactivity, learning/memory deficits, and social interaction abnormalities (Bolduc et al., 2008, 2010a,b; Coffee et al., 2010, 2012; Tessier and Broadie, 2012).

### CRITICAL PERIOD DEVELOPMENT OF E/I RATIOS IN ASDs

Autism spectrum disorders have been ascribed to altered E/I synaptic balance, which likely reflects defects in A-D synapse elimination/addition specific to different classes of synapse (Rubenstein and Merzenich, 2003; Hensch, 2005; Ramocki and Zoghbi, 2008; Gatto and Broadie, 2010). Neural circuits must carefully balance excitatory and inhibitory connections during critical period development, and theories of synaptic homeostasis posit that compensatory alterations prevent runaway signaling (Turrigiano and Nelson, 2004; Maffei and Fontanini, 2009). The appearance of altered E/I ratio is linked with critical periods of brain development, as runaway hyperexcitable circuits fail to mature properly without inhibitory input (Rubenstein and Merzenich, 2003; Rippon et al., 2007). In support of this idea, postmortem neocortex tissue from ASD patients shows reduced vertical arrays of glutamatergic and GABAergic mini-columns and disordered peripheral neuropil space (Casanova, 2006). Moreover, ASD postmortem studies reveal a reduction in glutamic acid decarboxylase (GAD), the rate-limiting enzyme in GABA synthesis (Fatemi et al., 2002). On the postsynaptic membrane, samples from ASD patient brains also contain reduced GABA-A receptor expression (Fatemi et al., 2009a,b). Crucially, excitatory circuits must be balanced by GABAergic inhibitory interneurons that form connections progressively during late-stage critical period development (Bacci et al., 2005). Competing theories of both hypoinhibition (GABAergic deficits) and hyperexcitation (excitatory excess) underlying autistic disease states are well known (Gatto and Broadie, 2010). However, many results support hypoinhibition models that complement hyperexcitation models; these models are not necessarily mutually exclusive and may represent two counterbalancing underpinnings of ASD disease states.

**FIGURE 2:**
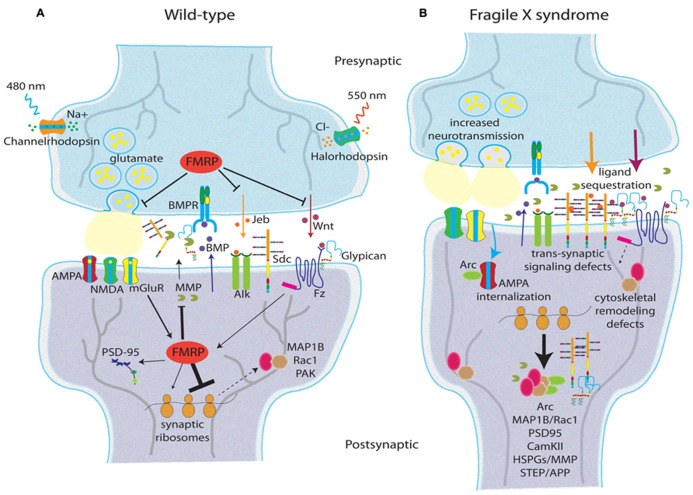
**Synaptic changes in the Fragile X syndrome disease state.** Schematic depictions of glutamatergic synapses in wild type **(A)** and FXS **(B)** conditions showing molecular changes in the response to activity. Data reflect human FXS patient studies, as well as mouse and *Drosophila* FXS animal models. **(A)** In wild-type, FMRP regulates glutamate release and anterograde *trans*-synaptic signaling (e.g., Jeb and Wnt), but does not demonstrably affect retrograde signaling (e.g., BMP; Friedman et al., 2013). FMRP acts as a translational repressor limiting synthesis of cytoskeletal regulators (e.g., MAP1B, Rac1, PAK; Zhang et al., 2001; Bongmba et al., 2011; Dolan et al., 2013), AMPA-R regulators (e.g., Arc; Costa et al., 2012; Niere et al., 2012), MMPs (Bilousova et al., 2009), and heparan sulfate proteoglycans (HSPGs: Dlp and Syd; Dani et al., 2012). FMRP is activity regulated downstream of mGluR signaling, but likely responds to other activity signals as well. **(B)** In the FXS state, lack of FMRP leads to increased neurotransmission, increased HSPG production (which in turn sequester anterograde *trans*-synaptic signaling molecules), increased MMP synthesis/activity, cytoskeletal remodeling defects, and changes in postsynaptic AMPA glutamate receptor cycling. Two engineered rhodopsin variants allow non-invasive modulation of neural activity: channelrhodopsin responds to 480 nm blue light to gate Na^+^ influx (depolarization), and halorhodopsin responds to 550 nm amber light to gate Cl^-^ influx (hyperpolarization; Fenno et al., 2011).

Fragile X syndrome is among the best-characterized ASD disease states. FMRP is found at the synapse, positivity upregulated by neuronal activity and regulates A-D processes including synapse elimination (**Figure 2**; Antar et al., 2004; Gabel et al., 2004; Tessier and Broadie, 2008; Wang et al., 2008a; Pfeiffer et al., 2010). FXS may be characterized by a failure to remove immature synaptic connections and properly balance E/I synapse ratio during critical period development (Comery et al., 1997; Irwin et al., 2000, 2001; Galvez et al., 2005; McKinney et al., 2005). For example, *Fmr1* KO mice display brain-region specific increases and decreases in GAD expression (D’Hulst et al., 2006; Adusei et al., 2010); increased in cortex, brainstem, and diencephalon (El Idrissi et al., 2005), and decreased in amygdala (Olmos-Serrano et al., 2010). FMRP also regulates GABA-A receptor expression (Liu et al., 2013), as *Fmr1* KOs show reduced GABA-R subunit mRNA (D’Hulst et al., 2006; Gantois et al., 2006) and protein (El Idrissi et al., 2005; Curia et al., 2009; Adusei et al., 2010). Recent work suggests that the timing rather than the absolute expression levels of GABA_A_Rs α1, α2, and gephyrin are altered in *Fmr1* KO mice (Kratovac and Corbin, 2013), once again supporting the theory of critical period dysfunction. It is now clear that GABAergic changes are regionally specific, as *Fmr1* KO mice display reduced inhibitory synapses within the basolateral amygdala (Olmos-Serrano et al., 2010), yet increased inhibitory synapses are noted in CA1 region of hippocampus (Dahlhaus and El-Husseini, 2010), providing direct evidence of E/I imbalance. *Fmr1*-deficient mice exhibit defects in GABAergic neocortical circuits (Selby et al., 2007), with differences in the neocortical E/I balance (Gibson et al., 2008). There is some conflicting data from functional GABAergic studies, with decreased tonic inhibition in recordings from subicular neurons (Curia et al., 2009), decreased tonic and phasic inhibition in the amygdala (Olmos-Serrano et al., 2010), and increased inhibitory transmission in striatal spiny neurons (Centonze et al., 2008) all reported in the mouse FXS model. A recent study reports a cell-specific presynaptic role for FMRP in excitatory neurotransmission onto inhibitory interneurons in layer 4 of mouse cortex (Patel et al., 2013), with mice mosaic for *Fmr1* displaying decreased glutamate release probability. This defect was not observed in neurotransmission between excitatory neurons, showing a cell-specific role for FMRP and a potential mechanistic basis for E/I imbalance in the FXS disease state.

The opposing side of the E/I ratio – excitatory signaling – is even better explored in ASD models, especially in the FXS disease state (Rubenstein and Merzenich, 2003). Excitatory neurons are intrinsically more excitable in *Fmr1* KO mice (Gibson et al., 2008), with elevated Ca^2^^+^ signaling (Goncalves et al., 2013) and excitatory networks that are structurally hyperconnected (although individual excitatory connections are slower; Testa-Silva et al., 2012). Excessive mGluR signaling, as a reporter of glutamatergic synapse activity state, is widely reported (Bear et al., 2004; Bear, 2005). The mGluR theory of FXS suggests that disease symptoms are due to exaggerated downstream consequences of aberrant mGluR1/5 signaling. Crucially, FMRP is locally synthesized in response to mGluR activation (Weiler et al., 1997), and mGluR-mediated hippocampal LTD is exaggerated in *Fmr1* KO mice (Huber et al., 2002; Nosyreva and Huber, 2006). FMRP is dephosphorylated (by PP2A) upon stimulation of group I mGluRs, which leads to a rapid increase in translation (Narayanan et al., 2007). In line with these studies, mGluR5 heterozygosity rescues many of *Fmr1* KO mice phenotypes (Dolen et al., 2007). Also, group 1 mGluR antagonists [e.g., MPEP (2-methyl-6-(phenylethynyl)-pyridine)] ameliorate several behavioral phenotypes in *Fmr1* KO mice (Yan et al., 2005; Choi et al., 2010b). Recent mechanistic studies show that mGluRA activation starts a cascade of events leading to FMRP phosphorylation and subsequent synthesis of Arc, and ultimately mGluR-associated LTD (Niere et al., 2012). In addition, activation of serotonin 7 receptors (5-HT7) can reverse mGluR-induced AMPA internalization in FXS model mice, effectively correcting mGluR-LTD (Costa et al., 2012). Although initial formation of auditory cortex is normal in *Fmr1* KO mice, mutants fail to undergo experience-dependent reorganization, suggesting an altered auditory critical period that is mGluR-dependent, as MPEP suppressed the sound-induced reorganization phenotype (Kim et al., 2013a). Sensory-dependent reorganization of auditory cortex has been explored at length, with A-D changes in hippocampus including neurogenesis, learning and memory, and neural connectivity (Chaudhury et al., 2013).

There are some caveats with the mouse FXS model. One issue is the timing of the FMRP loss, as in mouse models the *Fmr1* gene is deleted and therefore not expressed (Mientjes et al., 2006), whereas the human gene is silenced via methylation during embryonic development, but is expressed at early stages (Willemsen et al., 2002). Furthermore, human patients may display *Fmr1* mosaicism across cell types, due to methylation specificity or variable presence of a premutation (Stoger et al., 2011). Finally, mouse *Fmr1* phenotypic effects are often surprisingly mild, transient, and heavily dependent on genetic background (Spencer et al., 2011). Although the *Drosophila* FXS model does not address the first two issues, *dfmr1* null phenotypes are generally both more robust and more penetrant (Tessier and Broadie, 2012). Excitatory synaptic signaling (glutamatergic and cholinergic) pathways have both been extensively studied in the *Drosophila* FXS model (**Figure 2**). Electrophysiological studies indicate increased excitability, A-D synaptic vesicle cycling and neurotransmission in *dfmr1* null glutamatergic synapses (Zhang et al., 2001; Gatto and Broadie, 2008). *Drosophila* FMRP and sole mGluR (DmGluRA) display mutual feedback regulation, as FMRP expression increases with the loss of *dmGluRA*, and DmGluRA expression increases with loss of *dfmr1* (Pan et al., 2008). Crucially, many FXS phenotypes are ameliorated by feeding of mGluR antagonists (e.g., MPEP; McBride et al., 2005; Choi et al., 2011), and MPEP phenocopies the genetic loss of *dmGluRA* (Pan and Broadie, 2007; Pan et al., 2008). Importantly, *dfmr1*; *dmGluRA* double null mutants partially rescue excitatory defects witnessed under high frequency stimulation paradigms (Repicky and Broadie, 2009), providing a partial genetic basis for a hyperexcitable state in FXS. In the absence of FMRP, increased mGluR function leads to decreased cyclic AMP, which is further correlated with deficits in olfactory learning and memory (Kanellopoulos et al., 2012). Intriguingly, cAMP positively regulates transcription of *dfmr1*, via PKA and CREB (Kanellopoulos et al., 2012), thereby linking glutamatergic signaling and FMRP at the nucleus. In addition, recent work in our laboratory has identified alterations in the inhibitory circuitry of *dfmr1* null flies. *dfmr1* mutants are characterized by reduced GAD expression in the adult brain, developmental stage-specific dysmorphia in GABAergic axons innervating the MB calyx, and altered GABAergic Ca^2^^+^ dynamics (Gatto et al., 2014). This data importantly implicates altered inhibitory neurotransmission in the *Drosophila* model of FXS, and further validates the conservation of FMRP function in the fruit fly brain.

### DYSMORPHIC SYNAPSES IN ASD DISEASE STATES

Most ASD human studies on synaptic dysmorphia focus on the dendritic domain. For example, ASD patients display increased dendritic spine densities on cortical projection neurons (Hutsler and Zhang, 2010). FXS patients also display elevated spine density, with processes displaying elongated, “tortuous” structures (Irwin et al., 2001), perhaps suggestive of defects in synapse elimination. Similar dendritic dysmorphia are common in many other neurological disease states (Marin-Padilla, 1972; Purpura, 1974; Kaufmann and Moser, 2000), which may reflect developmental arrest or an attempted compensation for the lack of functionally mature spines (Fiala et al., 1998). In particular, it should be noted that these dendritic phenotypes are not restricted to the autism spectrum (sociolinguist deficits), as individuals with schizophrenia (perception deficits) and Alzheimer’s disease (memory dysfunction) also display abnormal dendritic spine architectures (Penzes et al., 2011). These three forms of neurological dysfunction are distinct in symptomology, yet this specificity may be rooted in the timing and onset of synaptic dysfunction (**Figure 1B**).

Protrusion dynamics are just as important for synaptogenesis in genetic model systems (Ziv and Smith, 1996; Luikart et al., 2008). In the mouse FXS model, for example, there is delayed functional spine formation in the hippocampus (Braun and Segal, 2000), and ultimately fewer spines with mature, bulbous morphology (Irwin et al., 2000). SD leads to changes in spine protrusion dynamics in neonatal mice (Lendvai et al., 2000), demonstrating A-D regulation. Specifically, *Fmr1* KO mice deficits may result from deficits in experience-dependent plasticity during critical periods of synaptic refinement (Dolen et al., 2007; Bureau et al., 2008). During *in vivo* time lapse imaging through cranial windows in neonatal mice, layer 2/3 neurons show a dramatic decrease in dendritic spine dynamics during the first 2 weeks as mushroom-shaped spines replace filopodia and protospines, whereas *Fmr1* KO mice show developmental delays in the downregulation of spine turnover and the transition to mature spines (Cruz-Martin et al., 2010). Importantly, mGluR blockage accentuated these phenotypes in *Fmr1* KO mice (Cruz-Martin et al., 2010), providing a different link to A-D synaptic remodeling in the FXS disease state.

The dynamic nature of dendrites enhances their ability to sample the extracellular space for suitable presynaptic terminals (Ziv and Smith, 1996; Bonhoeffer and Yuste, 2002). Immature synapses from as adhesions between dendritic filopodia and axons (Fiala et al., 1998). Perturbations in these dynamics lead to altered synaptogenesis, for example, as demonstrated in Ephrin B-deficient (Kayser et al., 2008) and neurotrophin-deficient (Luikart et al., 2008) conditions. Following initial contact, spine dynamics are necessary for A-D remodeling (Lendvai et al., 2000; Yuste and Bonhoeffer, 2001, Yuste and Bonhoeffer, 2004; Holtmaat et al., 2006), and then strongly downregulated at the end of the critical period of synapse selection (Ziv and Smith, 1996). In FXS animal models, the failure to stabilize dendritic spines in developmental critical periods suggests *Fmr1* null protrusions have problems maintaining proper balance between stability and dynamism (Antar et al., 2006; Pfeiffer and Huber, 2007), resulting in fewer mature synaptic connections (Cruz-Martin et al., 2010). FMRP presumably modulates synaptic stability through regulation of mRNAs coding for dendritic spine regulators (Bagni and Greenough, 2005; Bassell and Warren, 2008), such as the key postsynaptic scaffold PSD-95 (postsynaptic density protein 95) as one example (**Figure 2**; Zalfa et al., 2007). Recent reviews outline the spine dysmorphia in the mouse FXS model in more detail (He and Portera-Cailliau, 2013).

Differences in axonal development in the mouse FXS model have not been as extensively studied. However, FMRP is localized at axon growth cones, which are far less dynamic in* Fmr1* KO mice (Antar et al., 2006). More thorough studies in the *Drosophila* FXS model demonstrate progressive differences in axonal projection and synaptic process pruning in the central brain MB of *dfmr1* null mutants (Tessier and Broadie, 2012). The *Drosophila* model demonstrates defects in the development of neuronal architecture (Zhang et al., 2001) and inappropriate A-D pruning (Tessier and Broadie, 2009) throughout the brain. For example, the synapses of small ventrolateral neurons (sLNvS) in the circadian regulation circuitry are overelaborated in *dfmr1* nulls (Dockendorff et al., 2002; Gatto and Broadie, 2009), a developmental phenotype that can be rescued only during the late pupal/early adult critical period, but not in early pupal stages or in mature adult stages (Gatto and Broadie, 2009), demonstrating a transient critical period requirement for FMRP. In the MB circuit, FMRP is required to limit outgrowth during an early phase and to subsequently prune synaptic branches in a later phase, and both phases are dependent on activity input (Tessier and Broadie, 2008). Furthermore, MB neurons in *dfmr1* nulls demonstrate age-dependent increases in calcium signaling dynamics, as well as deficient expression of several calcium-binding proteins (Tessier and Broadie, 2011), suggesting activity is driving a calcium signaling cascade coupling structural and functional developmental changes in the FXS disease state. Collectively, these defects all map to the early-use, A-D critical period of synaptic remodeling.

### A-D TRANSLATION MISREGULATION IN THE ASD FRAGILE X SYNDROME

Autism spectrum disorders have been linked to hundreds of genes (Abrahams and Geschwind, 2008; Toro et al., 2010; Devlin and Scherer, 2012), and the number keeps jumping higher, with candidates in pathways affecting many distinct neuronal functions (Delorme et al., 2013). Importantly, however, many of these genes are modulated by neural activity, either directly or indirectly (Morrow et al., 2008; Chahrour et al., 2012; Ebert and Greenberg, 2013), and a number are known to be involved in A-D neural circuit modification (Toro et al., 2010). Rare *de novo* mutations have implicated a large network of genes directly involved in synaptogenesis and synaptic function (Gilman et al., 2011). Nevertheless, ASD modeling is made difficult by the underlying genetic diversity, and this difficulty is compounded by the array of symptoms described in human conditions and debates about appropriate genetic models (Crawley, 2007). The genetic bases of autism can be divided into a number of molecular groups: (1) chromatin remodeling, (2) cytoskeletal dynamics, (3) synaptic scaffolds, (4) neurotransmitter receptors and transporters, (5) second messengers, (6) cell adhesion molecules, and (7) secreted proteins (Persico and Bourgeron, 2006). Clearly the genetic basis of ASDs is massive area, and we focus here only on the FXS disease state, which may be an A-D translation control point for a number of these protein classes.

The monogenic FXS disease state (Verkerk et al., 1991) is typically caused by an unstable 5′ trinucleotide expansion in the promoter region of the *Fmr1* gene leading to hypermethylation and transcriptional silencing (Leehey et al., 2008). FXS patients exhibit delayed developmental trajectories, working memory deficits, circadian defects, hypersensitivity to sensory input, seizures, increased anxiety and hyperactivity (Harris et al., 2008), and a 30% comorbidity with autism (Zingerevich et al., 2009). Furthermore, FMRP may be associated with other neurological disease states, as schizophrenic patients have reduced FMRP in the periphery (Kovacs et al., 2013) and cerebellum (Fatemi et al., 2010), correlating with poor performance on perceptual integration tasks (Kelemen et al., 2013). The expanding web of FMRP associations (Bourgeois et al., 2009; Hagerman et al., 2010; Fatemi and Folsom, 2011) underlines the importance of this mRNA-binding translational regulator, with hundreds of candidate mRNA targets (Miyashiro et al., 2003; Darnell et al., 2011; Bagni et al., 2012). FMRP forms large cytosolic ribonuclear particles (RNPs), which are associated with mRNA transport, stability, and translation control (Bagni et al., 2012). RNP transport dynamics are altered in *Fmr1* KO mice, with reduced kinesin-associated mRNAs (Dictenberg et al., 2008). However, FMRP does not appear to be necessary for steady-state maintenance or constitutive localization of the majority of its target mRNAs (Steward et al., 1998; Dictenberg et al., 2008), although direct measurement of protein synthesis in hippocampal slices (Dolen et al., 2007), hippocampal culture (Osterweil et al., 2010), and synaptosomes (Muddashetty et al., 2007) shows global elevations in the *Fmr1* KO mouse.

Fragile X mental retardation protein is such an important focus because it is poised to directly and quickly respond to activity changes at the synapse. FMRP transports mRNA within the neuron and specifically at synapses in an A-D manner via association with microtubule-associated motor proteins (Kanai et al., 2004; Antar et al., 2005; Ferrari et al., 2007; Dictenberg et al., 2008; Charalambous et al., 2013). Targets of FMRP (Billuart and Chelly, 2003) include the small GTPase *Rac1* and its effector p-21 activated kinase (*PAK*), functioning as actin regulators (Bokoch, 2003). Rac1 is necessary for dendritic spine development, loss of FMRP leads to over-activation of Rac1 (**Figure 2B**), and Rac1 pharmacological inhibition leads to suppression of LTD in *Fmr1* KO mice (Bongmba et al., 2011). In addition, A-D stimulation of hippocampal synapses leads to increased phosphorylated PAK in wild-type, but not *Fmr1* KO mice, and mutants were unable to maintain actin cytoskeletal A-D changes (Chen et al., 2010). Moreover, small molecule inhibition of PAK suppresses *Fmr1* null phenotypes including dendritic spine morphology, seizures, hyperactivity, and repetitive movements (Dolan et al., 2013). Another target, PSD-95 is an adaptor protein associated with glutamatergic receptors (Sheng and Kim, 2002); mice deficient in PSD-95 show dendritic spine dysmorphia in striatum and hippocampus (Vickers et al., 2006). FMRP may regulate PSD-95 partially through stabilization of PSD-95 mRNA, a process that is enhanced with mGluR activation (**Figure 2A**; Zalfa et al., 2007). FMRP also associates with *futsch/MAP1B* mRNA, a microtubule regulator of synaptic growth (Roos et al., 2000), that can be localized at the growth cone of developing axons (Antar et al., 2006), and is localized within FMRP–RNP granules in cultured hippocampal neurons (Antar et al., 2005). FMRP and *futsch* associate in co-immunoprecipitation assays, and its expression is inversely correlated with FMRP expression (Zhang et al., 2001). Importantly, *futsch* loss of function corrects the synaptic overgrowth phenotype in *dfmr1* nulls (**Figure 2B**; Zhang et al., 2001). Application of the axon guidance molecule Semaphorin-3A (Sema3A) to hippocampal culture leads to MAP1B protein synthesis, but this response is attenuated in *Fmr1* null neurons (Li et al., 2009), thereby linking the activity of an axon guidance molecule with local FMRP-dependent translation. More recent work has shown that FMRP regulation of *futsch* is downstream of BMP and Spartin signaling (Nahm et al., 2013), thereby linking a key *trans*-synaptic signaling pathway with cytoskeletal changes in presynaptic neurons.

Several recent studies have provided new mechanistic insights about FMRP function at the synapse. Two recently identified FMRP targets are striatal-enriched protein tyrosine phosphatase (STEP) and amyloid precursor protein (APP), which appear to underlie at least a portion of mouse *Fmr1* phenotypes, as genetic reduction of either can suppress audiogenic seizure, anxiety, and social deficits in the disease model condition (Westmark et al., 2011; Goebel-Goody et al., 2012; **Figure 2**). Another target of FMRP is *Arc* mRNA (Zalfa et al., 2003), an A-D immediate early gene (Link et al., 1995) strongly linked to synaptic function (Park et al., 2008). Importantly, Arc protein functions to stimulate endocytosis of AMPA glutamate receptors (Chowdhury et al., 2006), an action correlated with the A-D induction of LTD (Park et al., 2008). FMRP plays an important modulatory role in this A-D process, acting as a translational repressor of *Arc* synthesis during mGluR-LTD (Niere et al., 2012). FMRP also regulates several potassium ion channels. For example, FMRP interacts with Kv3.1b mRNA in brainstem synaptosomes, and the A-D increase in Kv3.1b channel expression in wild type mice is abolished in *Fmr1* null mice (Strumbos et al., 2010). Moreover, FMRP can directly interact with potassium channel proteins to regulate channel kinetics, including Slack channels (Brown et al., 2010) and the β4 subunit of BK channels (Deng et al., 2013). Therefore, FMRP can no longer be described as solely a translational regulator, with protein–protein interactions regulating excitability demonstrating an additional vital role for FMRP function.

The final area of focus for FMRP regulation lies in the extracellular space. Both mouse and *Drosophila* FXS models have recently been documented to show large increase in the levels of synaptic matrix metalloproteases (MMPs), a family of extracellular proteases involved in synaptic development, function, and plasticity (**Figures 2A,B**; Rivera et al., 2010). MMPs cleave secreted as well as membrane-anchored proteins during synaptogenesis and A-D synaptic remodeling (Ethell and Ethell, 2007). Specifically, MMP-9 expression and activity are increased in *Fmr1* KO mice (Bilousova et al., 2009), with MMP-9 locally translated at synaptodendritic domains in an A-D manner (Dziembowska et al., 2012). MMP-9 mRNA is transported and regulated by FMRP, and increased MMP-9 expression is found in *Fmr1* null synaptosomes from mouse hippocampus (Janusz et al., 2013). Crucially, a drug inhibitor of MMPs, minocycline, effectively restores synaptic architecture and behavioral defects in the mouse FXS model (**Figure 2**; Bilousova et al., 2009). The mechanistic link between MMPs and ASDs may lie with MMP substrates, especially the HSPGs, which are well-established proteolytic targets of MMPs (Choi et al., 2012). HSPGs bind a variety of molecules, including growth factors, morphogens, and cell surface receptors, effectively modulating a hosts of biological functions (Bishop et al., 2007). In BTBR autism mouse models (Scattoni et al., 2011), hippocampal sclerosis (HS) immunoreactivity is reduced (Meyza et al., 2012). Conditional inactivation (neuronal specific) of *Ext1*, an essential enzyme for HS synthesis, leads to defective glutamatergic neurotransmission and behavioral abnormalities similar to autistic phenotypes (Irie et al., 2012). Disruption of HSPGs in *Drosophila* leads to *trans*-synaptic signaling defects at the NMJ (Dani et al., 2012), causing impairments of synaptic structural and functional development. Moreover, in the *Drosophila* FXS model, both MMP mutation and the minocycline MMP inhibitor (MMPIs) treatment effectively suppresses synaptic architecture defects in motor neurons, clock neurons, and neurons of the central brain MB learning/memory center (Siller and Broadie, 2011). Perhaps linking these two mechanisms, the *Drosophila* FXS model displays dramatic upregulation of synaptic HSPGs (**Figure 2**), including a GPI-anchored Glypican and transmembrane Syndecan (Sdc; Friedman et al., 2013). These elevated co-receptors in turn inappropriately sequester Jelly Belly (Jeb) and Wnt Wg *trans*-synaptic signaling ligands to alter intercellular communication between pre- and postsynaptic cells during synaptogenesis (**Figures 2A,B**). Genetic correction of the synaptic HSPG upregulation in *dfmr1* null mutants corrects both structural overelaboration and elevated neurotransmission (Friedman et al., 2013), demonstrating this signaling mechanism to be causative to A-D synaptogenic defects in this FXS disease state model. Based on these extensive studies in mice and flies, MMPIs are currently in development for FXS therapeutics as discussed below.

### ASD THERAPEUTIC AVENUES

The A-D model of ASDs, especially as it applies to the regulation of critical period development of appropriate E/I synaptic ratios, suggests a number of therapeutic strategies. For example, the overabundant mGluR signaling theory underlying FXS dysfunction is supported by numerous mGluR mutant studies in mouse and *Drosophila* animal models (Bear et al., 2004, 2008; Bear, 2005; McBride et al., 2005; Dolen et al., 2007, 2010; Pan and Broadie, 2007; Dolen and Bear, 2008; Pan et al., 2008; Repicky and Broadie, 2009). Importantly, mGluR antagonists (such as MPEP) effectively rescue many of the mouse and *Drosophila* FXS model cellular and behavioral defects associated with FXS (McBride et al., 2005; Pan and Broadie, 2007; Bolduc et al., 2008; Dolen and Bear, 2008; Choi et al., 2010a; Dolen et al., 2010). MPEP is not available for human use due to toxicity, but new generation mGluR antagonists are in development (Levenga et al., 2010; Wang et al., 2010b; Pop et al., 2013). For example, chronic application of the mGluR antagonist CTEP suppresses learning and memory deficits and leads to regional improvements in brain function in *Fmr1* KO mice, as determined by perfusion imaging as an indirect measure of neural activity (Michalon et al., 2014), although it is important to note that CTEP also affected wild-type learning and memory. This first use of functional imaging in a mouse ASD model is an important step forward. Current FXS patient clinical trials include other mGluR antagonists (e.g., mavoglurant; Gantois et al., 2013) as well as GABA-B receptor agonists (e.g., arbaclofen; Henderson et al., 2012). Fenobam, a selective mGluR antagonist, improved prepulse inhibition in 6 of 12 FXS patients (Berry-Kravis et al., 2009). mGluR reverse agonists in phase II and III clinical trials were recently extended to younger children (Levenga et al., 2010), recognizing the early developmental focus likely necessary for effective intervention. For illustration, the rescue of spine morphology in cultured neurons from *Fmr1* KO mice by mGluR blockage is effective in young neurons but less so in older neurons (Su et al., 2011). Thus, it is important for interventions to target A-D critical periods.

On this opposing side of the E/I balance lies the therapeutic potential to increase GABA levels or potentiate GABA receptors, with the goal to alleviating FXS symptoms of hypoinhibition (Paluszkiewicz et al., 2011; Coghlan et al., 2012). Altered GABAergic inhibition is a common thread in many neurodevelopmental disorders and represent an important focus for therapeutics. Pharmacological approaches to GABAergic modulation address several components of inhibitory neurotransmission, including the GABA reuptake blockers Riluzole (Mantz et al., 1994; Jahn et al., 2008; Erickson et al., 2011b) and Tiagabine (Nielsen et al., 1991), GABA_A_R activators Ganaxolone (Biagini et al., 2010; Reddy and Rogawski, 2010) and Gaboxadol (Deacon et al., 2007; Lundahl et al., 2007; Olmos-Serrano et al., 2010), GABA_B_R activator Arbaclofen (Pacey et al., 2009), and Vigabatrin, an inhibitor of GABA catabolism (French et al., 1996; Coppola et al., 1997). Acamprosate is also interesting, as a drug that both antagonizes mGluR5 (Blednov and Harris, 2008) and agonizes GABA_A_Rs (Mann et al., 2008). A small uncontrolled trial with three adult FXS patients showed improvement in social behavior and communication after 16–28 weeks on acamprosate (Erickson et al., 2010), and similar gains in social communication were found in a small uncontrolled sample of autistic children (Erickson et al., 2011a).

Alternative pharmaceutical approaches to FXS focus on MMP and perhaps HSPG function in the synaptomatrix (Siller and Broadie, 2011; Dani and Broadie, 2012; Dani et al., 2012; Friedman et al., 2013). One obvious approach is the use of MMPIs, of which a large collection has been developed for human clinical trials on inflammatory and vascular diseases (Hu et al., 2007). For example, the tetracycline-derivative minocycline acting as an MMPI spurs maturation of hippocampal dendritic spines and represses anxiety and memory defects in the FXS mouse model (Bilousova et al., 2009), and similarly suppresses synaptic architecture defects in motor neurons, clock neurons, and MB learning/memory center neurons in the *Drosophila* FXS model (Siller and Broadie, 2011). The drug therefore offers a directed approach toward deficits in A-D mechanisms at the synapse as it aims to correct overactive MMP in the absence of FMRP. Minocycline has previously been successful in treating a variety of neurological disorders, including multiple sclerosis, Huntington’s disease, Parkinson’s disease, and Alzheimer’s disease (Wang et al., 2003; Choi et al., 2007; Kim and Suh, 2009). Minocycline treatments led to a long-term reduction in hyperactivity and audiogenic seizures in young, but not old mice (Dansie et al., 2013). In human trials, minocycline led to improved language, behavior, and attention in FXS patients in an uncontrolled study (Utari et al., 2010), and a recent 3-month double-blind, placebo-controlled trial with young FXS patients showed improvements in anxiety, mood, and clinical global impression (CGI) of FXS individuals given minocycline (Leigh et al., 2013). The mechanistic link between minocycline and MMPs in human patients has also been explored, as reduced MMP-9 activity correlates with CGI improvements in FXS patients (Dziembowska et al., 2013). Finally, the PAK-associated cytoskeletal changes in FXS models have been pharmaceutically targeted, with significant suppression of FXS phenotypes in the *Fmr1* null mouse (Dolan et al., 2013). There may be a clinical path forward targeting PAK and/or downstream cytoskeletal perturbations.

A quite different avenue for ASD treatment targets A-D critical period development via environmental enrichment and training intervention following early diagnosis (Dawson et al., 2010; Woo and Leon, 2013). To illustrate the impact of environmental input on cognitive development, Romanian orphans who received limited sensory stimulation can have profound social and cognitive defects, and many develop post-institutional autistic syndrome (Hoksbergen et al., 2005), suggesting that an ASD-like state can be achieved through purely environmental impoverishment. Thankfully many of these children make significant cognitive and social gains upon adoption and placement into enriched environments (Nelson et al., 2007). Importantly, enriched environments increase sensory input activity and have profound effects on A-D synaptic dynamics (Greenough et al., 1985; Beaulieu and Colonnier, 1987; Moser et al., 1995). In animal models, FXS mutant mice display a host of striking improvements when reared in enriched environments, including greater spine hippocampal spine density (Lauterborn et al., 2013) and improved spike timing long-term potentiation (LTP; Meredith et al., 2007). As a group of syndromes with strong links to A-D synaptic developmental defects, enrichment approaches are popular in ASD treatments (Reichow and Volkmar, 2010; Warren et al., 2011). For example, a peer-mediated theater-based intervention strategy for ASD children showed significant gains in core social deficits (Corbett et al., 2013). Similarly, a recent 6-month controlled trial showed significant gains in autistic children who underwent sensorimotor enrichment through olfactory and tactile stimulation and exercises for other cross-sensory stimulation (Woo and Leon, 2013). These behavioral intervention strategies are, crucially, focusing on multisensory domains and may enable great improvements in both social and cognitive abilities of ASD children.

## PART 3: *IN VIVO* MANIPULATION/READOUT OF ACTIVITY-DEPENDENT CHANGES

The recent emergence of tools to non-invasively drive and monitor neural activity represents a pioneering step forward for A-D neurodevelopmental studies. Compared to the relatively narrow and invasive strategies of traditional electrophysiology (Biran et al., 2005; Bjornsson et al., 2006), new optogenetic techniques provide simultaneous access to groups of neurons, which can be selectively targeted with a range of transgenic drivers (Kim and Jun, 2013). On the one hand, new optogenetic techniques using engineered rhodopsin variants have enormously advanced our ability to control activity with pulsed application of specific wavelengths of light in defined populations of neurons (Fenno et al., 2011). On the other hand, optical recording techniques, such as calcium sensors and voltage-sensitive fluorescent reporters, provide individual and massed readouts of neural activity throughout the imaged circuitry, albeit at a cost in sensitivity and temporal resolution compared to electrophysiological recordings (Grienberger and Konnerth, 2012; Mutoh et al., 2012). Thus, these new transgenic techniques provide unprecedented abilities to both drive and record *in vivo* neural activity, and therefore show great promise for the systematic dissection of A-D critical period mechanisms at the heart of ASD disease states.

Techniques for detecting neural calcium flux have been progressing for decades (Shimomura et al., 1962; Tsien et al., 1985), taking advantage of Ca^2^^+^ dynamics as a readout of neural activity (Berridge et al., 2003). Resting Ca^2^^+^ concentrations in neurons are typically <100 nM and rise 10- to 100-fold following a single action potential (Berridge et al., 2000), providing the ability to monitor spike number, timing, frequency, and levels of synaptic input (Yasuda et al., 2004). Genetically encoded calcium indicators (GECIs), such as the frGECIs and GCamps (Kotlikoff, 2007), have revolutionized *in vivo* Ca^2^^+^ imaging. The latest generations of GCamp sensors are especially exciting, as they display ultrasensitive kinetics and stably provide readout of neural activity over extensive periods of time (Akerboom et al., 2012; Chen et al., 2013). For instance, GCamp6 effectively records neural activity from large groups of neurons to small synaptic compartments, in animals ranging from *Drosophila* to mice (Chen et al., 2013). The recent development of red-shifted GECIs reduces tissue scattering, phototoxicity, and background fluorescence, and allows the simultaneous use of other tools, such as ChR2, which is activated with 480 nm light and would therefore overlap and interfere with traditional green fluorescent protein (GFP)-based calcium sensors (Akerboom et al., 2013). The tandem development of multiphoton imaging and GECIs now provide exquisite access for activity monitoring.

Although Ca^2^^+^ imaging serves as readout for diverse forms of A-D changes (e.g., depolarization, influx, store release, second messenger cascades), the direct detection of changes in membrane voltage would represent a more specific, direct readout of electrical activity. Small fluorescent hydrophobic dyes have long been able to detect changes in membrane potential, and can also be used to characterize propagation of electrical signals through a given circuit (Salzberg et al., 1973). However, these dyes have low penetrance to deep areas of the brain, and are not genetically targetable. Early generations of genetically encoded voltage indicator proteins (GEVIs) overcame some of these limitations (Siegel and Isacoff, 1997; Barnett et al., 2012), but failed to reliably demonstrate robust signals in intact animals. In contrast, the recently developed ArcLight system is a voltage sensor probes (VSP)-based fluorescent voltage sensor with greatly increased signal-to-noise ratio (Jin et al., 2012). In intact *Drosophila* brains, the ArcLight system effectively reports both action potentials and subthreshold events, demonstrating beautiful sensitivity (Cao et al., 2013). The *in vivo* applications for this new tool are exciting. For example, ArcLight has provided the ability to record rhythmic activity in *Drosophila* clock neurons, which have so far been inaccessible to electrophysiology approaches (Cao et al., 2013). Thus, GEVIs and GECIs are exciting new tools that grant non-invasive and increasingly penetrant representations of *in vivo* neural activity.

### OPTOGENETIC CONTROL OF NEURONAL ACTIVITY

Optogenetic techniques have revolutionized the ability to direct and dissect A-D processes during critical period development (Williams and Deisseroth, 2013). Delivery of engineered rhodopsin variants into targeted neurons provides a non-invasive means to control neuronal firing rates via pulses of specific wavelengths of light. ChR2 facilitate depolarization of the membrane by gating influx of Na^+^ ions when illuminated by blue light (see **Figure 2A**; Fenno et al., 2011). Conversely, halorhodopsins (eNpHR) respond to amber light by mediating Cl^-^ ion influx, thereby hyperpolarizing the membrane and inhibiting firing (**Figure 2A**; Zhang et al., 2007; Gradinaru et al., 2008). To date, most optogenetic studies have been either electrophysiological or behavioral work in mature animals including, for example, an alternative to deep brain stimulation in Parkinson’s disease model mice (Gradinaru et al., 2009), reduction of anxiety (Tye et al., 2011), serotonergic modulation of behavior (Warden et al., 2012), dopaminergic depression alleviation (Tye et al., 2013), and memory extinction (Van den Oever et al., 2013). However, developmental applications of optogenetic A-D modulation are beginning to appear. For example, ChR2-expressing *Drosophila* MB neurons respond to short blue light illumination (6 h) during critical period development with significant decreases in synaptic process branching, but this A-D synaptic pruning is completely abolished in FXS model animals (Tessier and Broadie, 2008, 2012). In mouse, ChR2 optogenetic stimulation causes a lasting increase in postsynaptic spine density and increased concentration of CaMKII, when paired with glutamate uncaging (Zhang et al., 2008). Furthermore, optogenetics is now capable of producing cortical maps, and can be coupled with Ca^2^^+^ imaging for readouts of activity alterations (Patterson et al., 2013). In addition, the coupling of optogenetic stimulation and immediate early active gene *NPAS4* mRNA facilitates identification of transfected neurons in mice (Bepari et al., 2012). The capacity to manipulate A-D neuronal structure and function *in vivo* in a developmental context is simply remarkable, and recent studies highlight the promise of these rapidly evolving techniques.

In the past year, optogenetics techniques have particularly begun to blossom. For example, delivery of ChR2 to thalamic neurons was recently shown to effectively silence cortical seizures in mice (Paz et al., 2013). Conversely, expression of eNpHR in hippocampal neurons also provided protection against seizures (Sukhotinsky et al., 2013). Halorhodopsin channels can also provide *in vivo* inhibition of motor activity (Liske et al., 2013). The emergence of red-shifted GECIs and optogenetic channels now facilitates dual transgenic manipulation and functional readout in the same animal (Akerboom et al., 2013). Combinatorial dye labeling can also facilitate the simultaneous detection of structural and functional changes without the use of genetics (Siegel and Lohmann, 2013). The development of optochemical G protein-coupled receptors (GPCRs), including light-agonized mGluRs, have been shown to be fast, bistable means to effectively suppress excitability and inhibit neurotransmitter release both in brain slices and *in vivo* (Levitz et al., 2013). The InSynC (inhibition of synapses with chromophore-assisted light inactivation) technique has been developed to directly inhibit neurotransmitter release, via a genetically encoded singlet oxygen generator (miniSOG) fused to two synaptic proteins, vesicle-associated membrane protein 2 (VAMP2) and synaptophysin (Lin et al., 2013b). Multiphoton uncaging of glutamate and GABA analogs has been demonstrated on individual dendritic spines in hippocampal slices (Hayama et al., 2013). Red-shifted excitatory optogenes (ReaChR) have improved membrane trafficking, higher photocurrents and faster kinetics, scatter less in passing through the tissue, and have been capable of driving action potentials in awake mice with illumination through the intact skull (Lin et al., 2013a).

Despite the justifiable excitement, it is important to note also important caveats to optogenetic applications, particularly for the use in A-D developmental studies. First, it was recently reported that long-term expression of ChR2 can alter axonal morphology in mice (Miyashita et al., 2013). In addition, high-level, long-term expression of fluorescent probes can lead to structural artifacts and phototoxicity (Packer et al., 2013). Second, optogenetics are generally delivered through viral means in mammalian models, and it has recently been reported that adeno-associated viruses (AAVs) can form tissue deposits after injection, which can lead to continued infection over time and alterations in the expression of the optogene (Packer et al., 2013). This problem may be avoided with single cell electroporation (Judkewitz et al., 2009), but this limits the applications. Third, extreme stimulation can lead to an exhaustion of synaptic transmission (Kittelmann et al., 2013). Therefore, it is important to modulate the expression and long-term, high frequency use of optogenes, and to use appropriate controls to detect any artifacts. Alternative approaches to optogenetics include the use of genetically targeted TrpA1 channels, which are a class of temperature-gated excitatory cation channels for depolarizing neurons (Viswanath et al., 2003; Dhaka et al., 2006; Hamada et al., 2008). Work in *Drosophila* illustrates the power of TrpA1 manipulations, specifically in comparison to ChR2 optogenetics, as neurons expressing TrpA1 can show stronger and longer lasting electrophysiological effects (Pulver et al., 2009).

### *Drosophila* METHODS FOR STUDYING A-D DEVELOPMENTAL MECHANISMS

The “relative” simplicity of the *Drosophila* brain (hundreds of thousands of neurons) provides a high level of understanding about clonal lineages and connectivity among small, defined populations of neurons, even down to the individually identified single neuron level (Chou et al., 2010; Chiang et al., 2011; Lovick et al., 2013; Takemura et al., 2013; Wong et al., 2013). Importantly, *Drosophila* genetics allows precise delivery of transgenic tools to these targeted neuronal populations using the GAL4/UAS bipartite system (Jones, 2009), and with the MARCM technique for clonal analysis down to the individually identified single neuron level (Lee and Luo, 2001; Ostrovsky et al., 2013). Our lab has utilized these techniques particularly to analyze individually identified neurons in the FXS disease model (Pan and Broadie, 2007; Tessier and Broadie, 2008; Gatto and Broadie, 2009; Siller and Broadie, 2011), as our best-defined *Drosophila* ASD model. More recently, *Drosophila* has gained an expansive catalog of highly selective neuronal GAL4 drivers, which utilize limited regulatory sequences for exquisitely limited expression (Jenett et al., 2012; Manning et al., 2012). These new tools are providing the capacity to target defined neuronal circuits within the *Drosophila* brain at a never-before achieved level of resolution. The use of selective drivers for high-resolution morphological readouts of individual neurons, in combination with non-invasive methods of activity modulation, will greatly enhance our understanding of A-D mechanisms of synapse remodeling. We predict that the utilization of these new classes of neural drivers (in MB, projection neuron, fan body, ellipsoid body, retina, etc.), in combination with both optogenetic and alternative activity modulation techniques, will provide much better dissection of neuron class-specific A-D developmental mechanisms within the next few years. These studies will lead to a deeper understanding of ASD model disease states within precise maps of brain circuits, such as the MB (Parnas et al., 2013; Perisse et al., 2013) and antennal lobes (Tanaka et al., 2012), which will provide a foundation for deciphering the molecular genetic bases of these disease states and engineering effecting treatment strategies.

What are the current limitations on this use of the *Drosophila* system? For optogenetics, *Drosophila* requires an essential co-factor, all-trans retinal (ATR), for ChR2 activation (Schroll et al., 2006; Ataman et al., 2008), although this feature does provide a useful control. Moreover, fluorescent tags on optogenetic channels label targeted neurons to grant cell-autonomous morphological readouts of A-D modifications, but fail to illuminate their synaptic partners, although clever mapping techniques are being developed [e.g., Genetic Reconstitution Across Synaptic Partners (GRASP); Feinberg et al., 2008]. For example, the GRASP technique was recently used to synaptically link MB Kenyon cells with modulatory aminergic neurons (Pech et al., 2013). In a similar fashion, the CaLexA (calcium-dependent nuclear import of Lexa) neural tracing method may be useful for mapping synaptic partners (Masuyama et al., 2012). Alternatively, synaptic mapping studies may be more suited for larger subsets of neurons (broader GAL4 drivers), which are coupled to known targets and could then be assayed through standard immunohistochemistry. Further downstream, the use of immediate early neural genes, may help illuminate downstream effects of activity modulation (e.g., *Dhr38*; Fujita et al., 2013). Importantly, use of the multiple methods now available for A-D manipulations during neurodevelopmental studies (e.g., ChR2, eNpHR, TrpA1, NaChBac) in the *Drosophila* FXS model can be used to directly test whether targeted brain circuitry is capable of responding appropriately to activity during defined developmental critical periods. Moreover, the capacity to genetically target subsets of neurons may allow suppression of downstream ASD-related phenotypes. For example, in the *Drosophila* FXS model, hypoinhibition (e.g., reduced GABAergic input) might be suppressed by increased depolarization of inhibitory interneurons using a GAD:Gal4 driver crossed to ChR2, and hyperexcitation (e.g., elevated mGluR activity) could similarly be suppressed by selectively hyperpolarizing specific groups of glutamatergic neurons with halorhodopsin. Such studies in the particularly well-characterized *Drosophila* FXS model could ultimately lead to new intervention strategies in FXS patients and, by extension, the treatment of other patient groups suffering ASDs.

## Conflict of Interest Statement

The authors declare that the research was conducted in the absence of any commercial or financial relationships that could be construed as a potential conflict of interest.
